# Re-Exposure of a PD-1 Inhibitor After Previous Immune-Related Adverse Events

**DOI:** 10.3390/curroncol33040180

**Published:** 2026-03-24

**Authors:** Jana Burghaus-Zhang, Carsten Schulz, Egle Ramelyte, Joanna Mangana, Deniz Özistanbullu, Johannes Kleemann, Alexander Enk, Jessica C. Hassel

**Affiliations:** 1 Department of Dermatology and National Center for Tumor Diseases, University Hospital Heidelberg, 69115 Heidelberg, Germanyjessica.hassel@med.uni-heidelberg.de (J.C.H.); 2 Department of Dermatology, University Hospital Zürich, 8006 Zürich, Switzerland; 3 Department of Dermatology, Johann Wolfgang Goethe University, 60629 Frankfurt am Main, Germany

**Keywords:** immune-related adverse events, immunotherapy, skin cancer

## Abstract

Background: Programmed cell death protein (ligand) 1 (PD-(L)1) inhibitors are well established in the treatment of dermatological tumors. Mostly, they are well tolerated, but in about 9–21% of patients, grade 3/4 immune-related adverse events (irAEs) occur. As treatment options are limited, it is of interest to determine whether readministration of another or the same PD-(L)1 inhibitor is safe. Methods: This is a multicenter, retrospective study on patients with metastasized dermatological tumors who were retreated with either the same or a different PD-(L)1 inhibitor after the development of irAEs. The study was conducted at centers in Heidelberg, Zurich, and Frankfurt. Results: 22 patients were included between April 2020 and December 2022 with a median age of 71 years. A total of 13 (59%) patients were re-exposed with the same antibody and nine (41%) received a different PD-(L)1 inhibitor. Six (46%) of the patients who were re-exposed to the same antibody had an irAE, of which 67% were identical with the first. In patients receiving a different PD-(L)1 inhibitor, four (44%) developed an irAE, of which 75% were identical with the first. Conclusions: Both an intraclass switch of PD-(L)1 inhibitor treatment and re-exposure with the same antibody after an irAE can be considered as options with a fair chance of improving therapy tolerance.

## Simple Summary

Given the limited treatment options for dermatological tumors and the increasing successful use of immunotherapies, immune-related adverse events are becoming a highly discussed focus of attention. In about 9–21% of patients who received a PD-(L)1 inhibitor grade 3/4 immune-related adverse events (irAE) occur, which often leads to avoidance of further immunotherapies in these patients. To evaluate the safety of readministration of another or even the same immunotherapy we retreated 22 patients with either the same (*n* = 13) or another (*n* = 9) PD-(L)1 inhibitor after development of an immune-related adverse event. Furthermore, 46% of the patients who were re-exposed with the same antibody and 44% of patients receiving a different PD-(L)1 inhibitor developed an irAE. Therefore, both a switch of PD-(L)1 inhibitor treatment and a re-exposure with the same antibody after an immune-related adverse event can be considered with a fair chance of improving tolerance.

## 1. Introduction

For systemic therapy of metastatic melanoma, immune checkpoint inhibitors (ICIs) are indispensable. Here, cytotoxic T-lymphocyte-associated antigen 4 (CTLA-4) and programmed cell death protein (ligand) 1 (PD-(L)1) inhibitors significantly prolong the progression-free and overall survival. Both the CTLA-4 and PD-1 pathways represent major immune checkpoint regulators, and their ligands are frequently expressed in melanoma. While the goal of therapy is to activate the immune system against the tumor, immune-related adverse events (irAEs) may occur as unintended side effects and can manifest in virtually any organ. However, the frequency of the most common irAEs differs between CTLA-4 and PD-(L)1 antibodies [1,2,3,4,5,6,7,8,9,10,11,12].

It is known that melanoma patients, who have major toxicity during therapy with ipilimumab, are able to tolerate PD-1 inhibitor treatment. In a study of our own group, ten patients safely received pembrolizumab after experiencing severe irAEs with ipilimumab therapy [13].

Even though PD-1 inhibitor therapies are generally well tolerated and the majority (80%) of the adverse events are grade 1–2 in severity, 9–21% of patients still suffer from grade 3/4 adverse events. A total of 3–8 patients out of 100 have to discontinue therapy due to any-grade adverse events [1,8,14,15,16]. The four most common immune-related adverse events associated with PD-1 antibody therapy are rheumatic (12%), thyroid (11%) colitis (7%), and hepatitis (3%) [4]. In contrast, ipilimumab therapy is more frequently associated with ir-colitis than thyroid or rheumatic adverse events [3,4].

Interestingly, the occurrence of rheumatic and thyroid adverse events during PD-1 inhibitor therapy has been associated with improved overall survival [4].

Comparing different PD-1 inhibitors, the number of irAEs with nivolumab, pembrolizumab and cemiplimab seem similar [16]. However, there are currently no data comparing the toxicity of different PD-(L)1 inhibitors within the same patient. The aim of this retrospective study was to analyze the frequency, severity and quality of irAEs in patients who experienced an irAE during a PD-1 inhibitor therapy and were then rechallenged with the same or another PD-(L)1 inhibitor in clinical routine.

## 2. Patients and Methods

This is a multicenter, retrospective study. Data from tumor patients from three different dermatological clinics in Heidelberg, Zurich and Frankfurt, Germany, were collected from April 2020 to December 2022.

Patients were included if they had histologically confirmed metastatic melanoma, cutaneous squamous cell carcinoma (cSCC) or Merkel cell carcinoma (MCC), and were treated with at least two different PD-(L)1 inhibitors or re-exposed to the same PD-1 inhibitor in clinical routine, regardless whether it was given in the adjuvant or advanced setting. In addition, eligible patients must have experienced an irAE during the first treatment. Patients receiving PD-1 inhibitor monotherapy as a direct continuation of CTLA-4/PD-1 inhibitor combined treatment were excluded with regard to the expected difficulty in separating the adverse events that are mainly caused by the recently applied CTLA-4 inhibitor.

Patient characteristics collected were age, lactate dehydrogenase (LDH) level at treatment start, the type, dosage und duration of PD-(L)1 inhibitor therapy, occurrence and grade of the irAE, usage of corticosteroids or other immunosuppressive agents to treat the irAE, reason for end of treatment and other therapies in between the different PD-(L)1 inhibitor treatments. In addition, response to therapy according to RECIST and progression-free survival was determined. All data were extracted from the patient’s medical records by the treatment center and then, after anonymization, were centrally collected and analyzed. The retrospective analysis of clinical data is approved by the ethical committee of the University Hospital Heidelberg (S-454/2015).

Patient characteristics were extracted into categorial and continuous variables. Different patient data were summarized using percentage or median ± interquartile range. For normally distributed data with even variance, the t-test was performed. Categorical data were tested for a significant connection using the X2-test. For non-normally distributed data, the Mann–Whitney-U-Test was performed. All analyses were performed using Microsoft Excel 2019.

## 3. Results

### 3.1. Patient and Treatment Characteristics

A total of 22 patients were included in this retrospective study, with a median age of 71 years (range of 38 to 83 years) (Table 1). More than 80% of patients were male (male:female = 18:4).

A total of 13 patients (59%) were rechallenged with the same PD-1 inhibitor (three with nivolumab, nine with pembrolizumab and one with avelumab) and nine patients (41%) received two different PD-(L)1 inhibitors. Among the latter group of patients, four were first treated with pembrolizumab and five started with nivolumab. Of the patients starting with pembrolizumab, two were subsequently treated with nivolumab and the other two with the PD-L1 inhibitor atezolizumab. All patients, who were first treated with nivolumab, received pembrolizumab as second-line therapy.

Of the 22 patients, 15 (68%) received the second PD(L)1 inhibitor without intervening therapy. The remaining patients received a median of one intervening therapy (IQR = 1).

The patients received various treatments: chemotherapy (*n* = 3), vaccination within a clinical trial (*n* = 3), BRAF/MEK inhibition (*n* = 1), ipilimumab monotherapy (*n* = 1), an epidermal growth factor receptor antibody (*n* = 1), intralesional Talimogene laherparepvec (T-VEC, *n* = 1), and ipilimumab plus nivolumab (*n* = 1).

The median time between the end of the first treatment and start of the second therapy was 32 ± 58 weeks for the patients who received the same PD-1 antibody and 23 ± 113 weeks for the group that was treated with a different PD-(L)1 inhibitor.

The PD-1 inhibitors were used in the approved dosages for melanoma. The dose of avelumab was 10 mg/kg bodyweight every two weeks; atezolizumab was applied either 1200 mg every three weeks or 840 mg every two weeks.

### 3.2. Immune-Related Adverse Events (irAEs)

Based on the inclusion criteria, all patients had an irAE during the first PD-1 inhibitor therapy. The most frequent irAE was ir-arthritis (*n* = 5), followed by ir-pneumonitis (*n* = 3) and rash (*n* = 3). Only five patients (23%) suffered from more than one irAE. Concerning severity, 15 irAEs (54%) were mild and 13 (46%) were of grade 3/4. On the whole, 14 (64%) patients were treated with systemic corticosteroids. A total of 50% of patients ended the first therapy due to an irAE. The other patients terminated the therapy mostly due to progression (*n* = 4) or complete response (*n* = 2).

**Figure 1:**
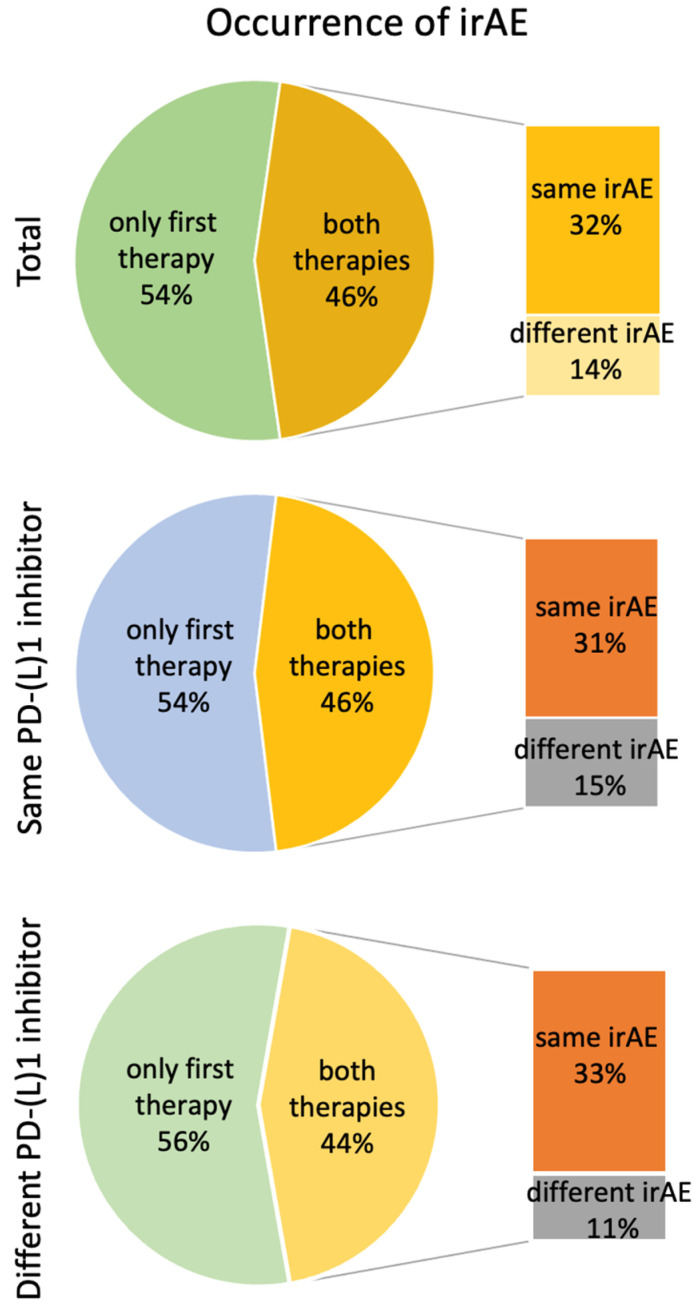
Reoccurrence of irAEs during second PD-(L)1 inhibitor treatment. *n* (total) = 22; *n* (same PD-(L)1 inhibitor) = 13; *n* (different PD-(L)1 inhibitor) = 9. irAE = immune-related adverse event.

All patients experienced an irAE during the first PD-(L)1 inhibitor therapy; Figure 1 illustrates how many patients also developed an irAE during the second therapy. The top pie chart (“Total”) shows the proportion of patients who experienced an irAE only during the first therapy or during both therapies, representing the recurrence rate of any irAE. The two lower pie charts show the same information for the subgroups of patients who received the same PD-(L)1 inhibitor twice and those who received two different PD-(L)1 inhibitors sequentially.

The adjacent bar charts further break down the proportion of patients with irAEs in both therapies according to whether the irAE during the second therapy was the same as during the first therapy or a different irAE.

The following specific results are shown: At re-exposure of any PD-(L)1 antibody, regardless of whether it was the same or a different one as before, 10 patients (46%) again developed an irAE. Seven of these had a relapse of the same irAE as before. Thus, 70% of the patients who experienced an irAE during the second therapy developed the same irAE as during the first treatment. This corresponds to 32% of the total study population, irrespective of whether an irAE occurred during the second therapy (Figure 1).

In the small subgroup of four female patients, two developed an irAE during the second therapy. In the subgroup of male patients, 44% developed an irAE during the second PD-(L)1 inhibitor therapy. There was no significant association between the patients’ sex and the occurrence of irAEs during the second therapy (*p* = 0.84).

The second therapy’s irAEs were not significantly more severe (median grade ± IQR therapy 1 = 2 ± 1.0; therapy 2 = 2 ± 1.3; *p* = 0.214). In particular, in all seven cases of recurrence of the same irAE, the severity grading was exactly the same as before.

**Figure 2:**
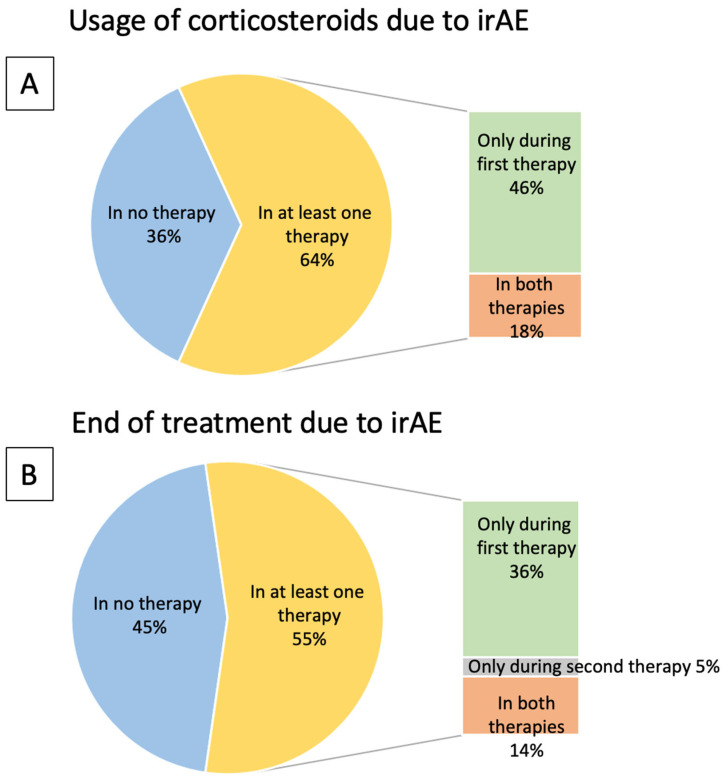
All patients, *n* = 22. (**A**) Differences in usage of corticosteroids during first and second PD-(L)1 antibody administration. (**B**) Frequency of termination of treatment due to adverse events during first and second PD-(L)1 inhibitor therapy. irAE = immune-related adverse event.

Figure 2 addresses whether patients received corticosteroids due to irAEs (Figure 2A) and whether therapy was discontinued because of adverse events (Figure 2B).

The pie chart in Figure 2A shows the proportion of patients who did not receive corticosteroids in either therapy versus those who received corticosteroids in at least one of the therapies. The adjacent bar chart further subdivides the group of patients who received corticosteroids in at least one therapy, according to whether corticosteroids were administered only during the first therapy or during both therapies. Since no patient required corticosteroids only during the second therapy, this subgroup is not shown. All percentages refer to the total study population. The detailed results are as follows:

A total of 64% of the patients received corticosteroids at least in one of the therapies. However, only four patients received corticosteroid treatment during the second PD-(L)1 inhibitor therapy (18%). Interestingly, these four patients had also received corticosteroids during the first treatment.

The pie chart in Figure 2B depicts the proportion of patients who did not discontinue any therapy due to irAEs versus those who discontinued treatment because of an irAE in at least one therapy. The adjacent bar chart further subdivides the group of patients who discontinued treatment because of an irAE in at least one therapy according to whether only the first therapy was discontinued due to irAEs, only the second or both. All percentages refer to the total study population. The detailed results are as follows:

The second PD-(L)1 inhibitor had to be terminated due to irAEs in only four cases. Three of these patients had also discontinued the first therapy because of an irAE, while only one patient had to stop the second therapy due to irAEs, despite not having discontinued the first treatment for the same reason (Figure 2).

Additionally, the recurrence of an adverse event was not significantly more often seen in patients who did not have an in-between therapy (43% vs. 47%). Furthermore, the time period before the start of the second therapy was not significantly shorter in patients who suffered from an adverse event during the second therapy (median time before second treatment: 30 weeks (irAE only during first therapy) vs. 32 weeks (irAE during both therapies), *p* = 0.500).

To evaluate if it makes a difference for treatment tolerance if you re-expose the patient to the same or a different PD(L)1 inhibitor, we then analyzed the patients separated in two groups, namely into patients who received the same PD(L)1 inhibitor twice (“same”) and patients who received two different ones (“different”).

#### 3.2.1. Rechallenge with the Same PD-(L)1 Inhibitor

Overall, 13 patients were re-exposed with the same PD-(L)1 inhibitor. In between the first therapy and the rechallenge, the patients either received another treatment (*n* = 3) or just had a treatment pause (*n* = 10). Three patients sequentially received nivolumab, one patient received the PD-L1 inhibitor avelumab, and the other nine patients were treated with pembrolizumab twice.

Even though all 13 patients had experienced an irAE during first administration of the PD-(L)1 inhibitor, only six patients (46%) suffered from an irAE during rechallenge (Figure 1). As displayed in Table 2, four patients suffered from the same irAE as during the first treatment: twice ir-arthritis and once ir-myocarditis and lichen planus. Four patients developed a different or additional new irAE.

Interestingly, we did not see a worsening of severity of irAE during the PD-(L)1 rechallenge. While 9 of the 16 reported irAEs during the first PD-(L)1 therapy were mild (grade 1/2, 56%), during second therapy, 63% of irAEs were classified as grade 1/2.

Eight patients (62%) were treated with corticosteroids for the irAE due to first administration of the antibody. At re-exposure, only three patients, who had already received corticosteroids during the first therapy, had to be treated with immunosuppressive therapy again (23%).

However, even though seven patients (54%) discontinued therapy due to an irAE after the first PD-(L)1 inhibitor, only three (23%) had to stop the rechallenge because of adverse events.

#### 3.2.2. Rechallenge with a Different PD(L)1 Inhibitor

Nine patients were retreated with a different PD-(L)1 inhibitor: seven of these received both nivolumab and pembrolizumab and two patients were treated with pembrolizumab followed by the PD-L1 inhibitor atezolizumab. All patients suffered from an irAE during the first therapy. Overall, half of the reported irAEs were mild and the other half were grade 3/4. Six patients (67%) had to be treated with corticosteroids. Termination of the first therapy due to an irAE was necessary in four cases (44%).

After the intraclass switch to another PD-(L)1 inhibitor, four patients (44%) again developed an irAE (Figure 1). Three of these experienced a recurrence of the same irAE (Table 2: ir-arthritis, ir-colitis, and rash). Only one irAE was classified as grade 3. This patient was also the only one who required treatment with corticosteroids after the intraclass switch. Only one patient discontinued the second treatment due to an irAE.

Notably, one of the two patients who were switched to a different PD-L1 inhibitor after treatment with pembrolizumab did not develop any adverse events under atezolizumab. This patient had previously experienced ir-arthritis during treatment with pembrolizumab and with ipilimumab plus nivolumab but remained asymptomatic under atezolizumab. The other patient experienced a recurrence of the previously observed ir-arthritis. However, during pembrolizumab treatment, this patient had also developed ir-nephritis and ir-colitis, which did not recur.

#### 3.2.3. Comparison of the Treatment Groups “Same” and “Different” PD-(L)1 Therapies

Regardless of whether the same PD-(L)1 inhibitor was used or whether an intraclass switch was performed, more than half of the patients had no adverse event during the second treatment. While this is already a great result, it is still of interest whether an intraclass switch should be performed. Therefore, the recurrence rate of an irAE was compared between the groups “same” and “different” and no significant difference was seen (*p* = 0.937). In the small subgroup of two female patients that developed an irAE during the second therapy, one had been treated with pembrolizumab after an irAE due to nivolumab. The other one was retreated with pembrolizumab. Also, in the subgroup of male patients, no relevant difference between the cohort that was retreated with the same (45%) or a different PD-(L)1 inhibitor (43%) was seen.

Additionally, there was no significant difference in the groups “same” and “different” regarding the necessity of corticosteroids during the second therapy (*p* = 0.474) and regarding the termination of treatment due to an irAE (*p* = 0.474).

Furthermore, the readministration of the same PD-(L)1 inhibitor did not lead to more severe irAEs in comparison to the intraclass switch (*p* = 0.665).

Concerning the types of irAE, irAEs that were likely to recur included ir-arthritis (*n* = 3), rash (*n* = 1), ir-colitis (*n* = 1), lichen planus (*n* = 1) and ir-myocarditis (*n* = 1) (Table 2).

Interestingly, efficacy was also similar between patients who received the same and those who received a different anti-PD(L)1 antibody. During the first anti-PD1 therapy, the response rate was 45%, the disease control rate was 95% and the median progression-free survival (PFS) was 14 ± 8 months. At re-exposure, 39% of patients who received the same PD-(L)1 inhibitor showed a response, while 22% responded after the intraclass switch (*p* = 0.421). Disease stabilization was achieved in 62% of the “same” group and 78% of the “different” group (*p* = 0.421). The median PFS during second treatment was 2 ± 5.25 months with the same antibody and 3 ± 2 months after the intraclass switch (*p* = 0.775).

## 4. Discussion

The safety of readministration of the same PD-(L)1 inhibitor has been addressed before in different cancer types, but a possible advantage of an intraclass switch has not been evaluated yet. As an example, Simonaggio et al. presented a cohort study including 40 cancer patients that were rechallenged with the same PD-(L)1 inhibitor. After readministration, 55% of the patients suffered from an irAE [17]. Santini et al. analyzed the same question for lung cancer patients and presented a similar amount of irAE recurrence (52%) after readministration. Interestingly, only half of the patients had a recurrence of the same irAE [18]. In our cohort of patients retreated with the same anti-PD1 antibody, 46% developed an irAE again, which is a slightly lower frequency. The rate of recurrence of the same adverse reaction was slightly higher (31%); however, all were in the same range.

These data are supported by a meta-analysis including studies with patients suffering from solid tumors, which were retreated with immune checkpoint inhibitors after discontinuation due to progression or adverse events, presented by Liu et al. In the subgroup of patients who ended the first ICI due to an irAE, 42,8% suffered from another adverse event during the rechallenge [19].

Nardin et al. also rechallenged patients with an immune checkpoint inhibitor after discontinuation, but the treatment pause was not related to an irAE, but to disease control. In these patients, an irAE after rechallenge occurred in 27%, which is slightly higher than the rate of irAEs for PD-(L)1 inhibitors in the literature [20].

Pollack et al. reported on the resumption of PD-1 inhibitor therapy after adverse events during treatment with ipilimumab/nivolumab. It has been shown that adverse events that are more frequent with ipilimumab (e.g., ir-colitis and ir-hepatitis) are less likely to recur [21]. In our cohort, ir-arthritis was the adverse event most likely to recur after resumption of PD-(L)-1 inhibitor therapy.

To avoid the recurrence of particularly severe irAEs, clinicians may be tempted to switch to a different anti-PD-1 antibody in the hope of achieving a slightly different adverse event profile. For anti-PD-1 monotherapy after ipilimumab–nivolumab combination therapy, this has been demonstrated [21]; however, this has not been shown for the use of different anti-PD-(L)1 antibodies within the same patient. In our small patient cohort, we found that an intraclass switch to another PD-(L)1 inhibitor was not safer than readministration of the same agent. Similarly, efficacy, as measured by response rate and progression-free survival (PFS), was comparable.

In contrast to this, Allouchery et al. observed less irAEs after readministration of the same ICI compared to a switch to another ICI. They reported patients with different kinds of cancer who were rechallenged with ICIs, including CTLA and PD-(L)1 inhibitors, after discontinuation of an ICI due to an irAE grade 2 or higher [22]. Importantly, the inclusion of combination therapies of CTLA and PD-(L)1 inhibitors allows for misinterpretation of the data, as the risk of suffering from an irAE after restarting a PD-1 inhibitor therapy is not comparable to the risk of an irAE during the initial combination therapy, even if the therapy was discontinued due to an irAE during the PD-1 inhibitor-only phase.

Theoretically, although interacting with the same checkpoint, different PD-1 inhibitors are not exactly the same. Lepir et al. explained the differences between the PD-1 antibodies by using different molecular protein–protein interactions [23]. In line with this, 30% of patients developed a different irAE compared to the irAE of the first anti-PD1 antibody. However, the same was true for retreatment with the same anti-PD1 and hence, cannot be explained by different antibody structures but potentially a different susceptibility of the patient for recurrence of a distinct irAE.

Most interesting is whether potentially lethal adverse events tend to recur. In our study, one patient developed ir-myocarditis and suffered from a relapsed myocarditis after readministration of immunotherapy. On the other hand, three patients suffered from ir-pneumonitis and none of them had a relapse. Nannini et al. summarized, in a systematic review, data on patients who had ir-aseptic meningitis due to either an ipilimumab/nivolumab combination or PD-(L)1 or CTLA4 inhibitor monotherapy. A total of 15 patients were rechallenged. Only one patient who was rechallenged with ipilimumab/nivolumab had a meningitis relapse. Two patients who were rechallenged with a PD-1 inhibitor monotherapy developed a new irAE [24].

Furthermore, it has already been discussed whether the switch to a PD-L1 antibody after an irAE can improve tolerance. A meta-analysis by Pillai et al. including data from 5744 non-small-cell lung cancer patients showed no significant difference in the number of adverse events between PD-1 and PD-L1 inhibitor therapy [25]. However, this does not give any information for individual patients. Unfortunately, due to low number of patients who were treated with a PD-L1 inhibitor, in the current presented study it was not possible to draw any conclusions.

To the best of our knowledge, this is the first retrospective study analyzing not only the safety of a rechallenge with a PD-(L)1 inhibitor after an irAE but also the possible im-pact of an intraclass switch of PD-(L)1 inhibition with the aim to improve therapy tolerance in dermatological tumor patients. However, we are very much aware of the limitations of this retrospective analysis with only a small number of patients, which can only be indicative that re-exposure to a different anti-PD1 antibody does not lead to better tolerability. Identification of a specific biomarker that identifies patients who have a greater probability of recurrence of an adverse event will be crucial in the future.

## 5. Conclusions

Both a readministration of the same PD-(L)1 inhibitor and an intraclass switch to another PD-(L)1 antibody can be safe with close surveillance. However, a switch to a different anti-PD(L)1 antibody seems not to improve tolerance in comparison to readministering the same. Therefore, a rechallenge with a PD-(L)1 inhibitor after a severe irAE can be considered in patients with limited treatment options to improve the clinical outcome of the tumor. These initial data need to be validated by a prospective, controlled trial.

## Figures and Tables

**Table 1:** Comparison of the patient characteristics between the different treatment groups: (A) all patients, (B) patients receiving the same PD-1 antibody twice, “same” (C) patients, receiving two different PD-(L)1 antibodies, “different”.

	All Patients	Same	Different	*p*-Value
Number of patients	22	13	9	
Male:Female	18:4	11:2	7:2	
Median age ± IQR	71.0 ± 22.8	75.0 ± 15.0	71.0 ± 23.0	0.560
Melanoma	19	10	9	
cSCC or MCC	3	3	0	
Median LDH start 1st PD-(L)1 inhibitor (U/L) ± IQR	263.0 ± 105.0	274.0 ± 71.0	238.0 ± 63.0	0.331
Median LDH start 2nd PD-(L)1 inhibitor (U/L) ± IQR	255.0 ± 69.0	285.0 ± 93.0	241.5 ± 46.5	0.058

IQR = interquartile range; cSCC = cutaneous squamous cell carcinoma; MCC = Merkel cell carcinoma; *p*-value regarding difference between treatment groups “same” and “different”.

**Table 2:** Specification of adverse events that only occurred during one therapy and those that reoccurred. In lines 3–21 of the first column, the different reported adverse events are listed. Columns 2–4 in the first row indicate whether the adverse event occurred both PD-(L)1 inhibitor treatments or only during the first PD-(L)1 therapy (column 2) or the second treatment. The columns in row 2 further specify whether the patient was retreated with the same or a different PD-(L)1 inhibitor. The numbers in columns 2–7 in rows 3–21 represent the number of patients who developed the adverse event indicated in the first column.

	Recurrence of irAE (No.)		Only in 1st Therapy (No.)		Only in 2nd Therapy (No.)	
	Same	Different	Same	Different	Same	Different
**ir-arthritis**	2	1	1	1		
**ir-nephritis**				1	1	
**rash**		1	1	1		
**ir-pancreatitis**			1	1		
**autoimmune bullous dermatosis**						1
**ir-pneumonitis**			2	1		
**ir-hypophysitis**				1		
**ir-diabetes**				1		
**ir-neuritis**			1	1		
**ir-colitis**		1	1			
**ir-thyreoiditis**			1			
**Psoriasis**					1	
**Polymyalgia rheumatica**			1			
**Lung fibrosis**			1			
**Vitiligo**			1			
**Lichen planus**	1					
**Urticaria**					1	
**ir-myocarditis**	1					
**ir-hepatitis**					1	

## Data Availability

The original contributions presented in this study are included in the article. Further inquiries can be directed to the corresponding author.

## Abbreviations

The following abbreviations are used in this manuscript:

PD-(L)1	Programmed cell death protein (ligand) 1
irAE	Immune-related adverse event
ICIs	Immune checkpoint inhibitors
CTLA-4	Cytotoxic T-lymphocyte-associated antigen 4
cSCC	Cutaneous squamous cell carcinoma
MCC	Merkel cell carcinoma
LDH	Lactate dehydrogenase
T-VEC	Talimogene laherparepvec
PFS	Progression-free survival
IQR	Interquartile range
